# Locally Differentially Private Heterogeneous Graph Aggregation with Utility Optimization

**DOI:** 10.3390/e25010130

**Published:** 2023-01-09

**Authors:** Zichun Liu, Liusheng Huang, Hongli Xu, Wei Yang

**Affiliations:** School of Computer Science and Technology, University of Science and Technology of China, Hefei 230026, China

**Keywords:** data privacy, local differential privacy, graph aggregation, heterogeneous graph

## Abstract

Graph data are widely collected and exploited by organizations, providing convenient services from policy formation and market decisions to medical care and social interactions. Yet, recent exposures of private data abuses have caused huge financial and reputational costs to both organizations and their users, enabling designing efficient privacy protection mechanisms a top priority. Local differential privacy (LDP) is an emerging privacy preservation standard and has been studied in various fields, including graph data aggregation. However, existing research studies of graph aggregation with LDP mainly provide single edge privacy for pure graph, leaving heterogeneous graph data aggregation with stronger privacy as an open challenge. In this paper, we take a step toward simultaneously collecting mixed attributed graph data while retaining intrinsic associations, with stronger local differential privacy protecting more than single edge. Specifically, we first propose a moderate granularity attributewise local differential privacy (ALDP) and formulate the problem of aggregating mixed attributed graph data as collecting two statistics under ALDP. Then we provide mechanisms to privately collect these statistics. For the categorical-attributed graph, we devise a utility-improved PrivAG mechanism, which randomizes and aggregates subsets of attribute and degree vectors. For heterogeneous graph, we present an adaptive binning scheme (ABS) to dynamically segment and simultaneously collect mixed attributed data, and extend the prior mechanism to a generalized PrivHG mechanism based on it. Finally, we practically optimize the utility of the mechanisms by reducing the computation costs and estimation errors. The effectiveness and efficiency of the mechanisms are validated through extensive experiments, and better performance is shown compared with the state-of-the-art mechanisms.

## 1. Introduction

Graph data are widely spread in people’s lives from policy formation and market decisions to medical care and social interactions, whose exploitation and utilization are crucial to improve the overall quality of data-driven services. However, the heavy dependence of these services on personal graph data brings up serious concerns about the abuses of their private information. In recent years, a number of organizations have been exposed for abusing and compromising personal data privacy [1,2,3], and these incidents have caused huge financial and reputational damage to both organizations and their users. In order to avoid these negative outcomes, some countries and regions have actively enacted relevant laws to provide legislative authorities for privacy protection, such as GDPR [4] and CCPA [5]. Therefore, devising privacy protection mechanisms, which reveal overall valuable statistical information without violating the privacy of individual data, has become a top priority for organizations and research fields nowadays.

Due to its rigorous theoretical guarantees, differential privacy (DP) [6,7] has become the de facto standard of privacy preservation. DP mechanisms utilize a centralized trustworthy data curator to collect individual private data, and ensure that the overall output statistics does not reveal individual private information by adding calibrated noise to aggregated results. As the scale of Web-enabled distributed devices grows, localized version of DP has been recently proposed to further reduce the risk of privacy breaches. Local Differential Privacy(LDP) [8] relies on no hypothetically trustworthy third-party data curator as in the conventional centralized DP, and provides on-device data perturbation and out-device purified statistics with rigorous privacy guarantee. Many companies have employed LDP based services, such as Apple [9,10], Google [11,12] and Microsoft [13].

LDP studies have been conducted in various fields, such as categorical data frequency publication [8,14,15,16] and numerical data mean estimation [13,17,18]. Subsequent research studies expand the scope to more complex data types, such as itemset release on set-valued data [19,20,21], decomposed distribution estimation on multidimensional data [22,23], and related data collection on key-value data [24,25]. However, studies on heterogeneous graph data are still scarce, which is a widely exploited data type in real-world applications, and data service providers wish to aggregate these heterogeneous graph data to analyze individual usage patterns and use them to improve the quality of services such as commodity recommendation [26,27], marketing [28] and pandemic tracking [29]. Consider heterogeneous social network as an example, each user (node) interacts through the social services belonging to multiple organizations or parties, and such communication linkages (edges) thus carry different numerical attributes, such as contacting frequencies and time intervals, and these attributes are potentially characterized as linkage weights. Users/organizations may also label part of edges as friendship, coworker-ship, kinship, political preference and sexual relationship. Accordingly, these attributed linkages represent the user engagement and usage frequency of corresponding social services, which is widely used in user profiling and recommendation systems. Another example is social–financial networks, where the users in one social network also have financial transactions. The social linkages between users may be attributed as friendship, coworkership and family, while the financial linkages between users contain fund transfer amount/time and trade amount/time. Aggregating the social–financial graph data is vital in marketing. As various social services provide location tracking systems, the so-called geosocial networks are also an important application of heterogeneous graphs. While part edges of the geosocial network are attributed social linkages, the geographical edges with trajectory distance and tracking time form a graph-based trajectory network. Combining and collecting these geosocial graphs provides significant pandemic tracking services.

Recently, graph data aggregation mechanisms under LDP constraint have been studied. By collecting perturbed degrees of pure graph data, Ref. [30] proposes to generate synthetic graph and [31] manages to aggregate subgraph statistics with extended privacy definition. Ref. [32] broadens the research scope to graph with node attributes. However, existing research studies mostly protect single edge privacy with edge-based LDP, while users in heterogeneous graph may require stronger privacy guarantee such as protecting a group of equally sensitive attributed edges from the statistical aggregation (e.g., protect all the sexual or political relations), and existing mechanisms may be insufficient to satisfy the potential heterogeneous graph privacy demands. Furthermore, existing mechanisms generally focus on single-attributed graph such as the weighted graph or the categorical-attributed graph, and leaves the heterogeneous graph aggregation challenge unresolved, which is to simultaneously collect mixed attributed graph data and intrinsic associations (between attributes and edges) while providing desirable utility.

In this paper, we take a step toward aggregating heterogeneous graph data with stronger local differential privacy protecting more than single edge. First of all, we characterize the two conventional variants of LDP definitionfor heterogeneous graph, integrate their characteristics and propose a fine-grained privacy definition with trade-offs between preservation strength and estimation accuracy. Under the moderate LDP definition, the problem of aggregating heterogeneous graph is addressed through two incremental stages, which are collecting categorical-attributed and heterogeneous graphs. For the former, we design a PrivAG mechanism to simultaneously sample and perturb subsets of encoded attribute and degree vectors, while retaining the relations reside within them. For the latter, we present an optimal binning scheme to segment and merge mixed attributed data, which serves as a preceding subtask for subsequent mechanism. Lately, we extend PrivAG mechanism to uniformly aggregate heterogeneous graph, and further devise optimization techniques targeting user-side randomization and server-side estimation, achieving better privacy–utility tradeoff.

The main contributions of this paper are summarized as follows:

- We propose an attribute-wise local differential privacy (ALDP) notion with moderate granularity between conventional node-based LDP and edge-based LDP, tradingoff privacy and utility between them, and formulate the problem of aggregating heterogeneous graph data under the ALDP notion as collecting attribute frequency and attribute-degree distribution.
- We apply padding and truncating for categorical-attributed graphs to handle the large data domain, and encode graph data as corresponding attribute and degree vectors. Then a utility-improved PrivAG mechanism is proposed to privately and simultaneously aggregate subsets of attribute and degree data.
- We present an adaptive binning scheme (ABS) to dynamically segment weighted edges and simultaneously collect mixed attributed data in the same process, reducing the computation cost to local devices and the estimation error caused by inconsistent distribution.
- We extend the privacy field to handle heterogeneous graphs and devise optimization techniques for user-side randomization and server-side estimation. The adaptive binning scheme and optimization techniques are integrated into the extended PrivHG mechanism.
- We validate the effectiveness and efficiency of our mechanisms based on extensive experiments, which are shown to have better performance than the state-of-the-art mechanisms.

The remainder of this paper is organized as follows. Section 2 introduces two conventional variants of LDP definition in graph data, and proposes a moderate attributewise local differential privacy. Section 3 formulates the problem of analyzing heterogeneous graph with ALDP and presents straightforward approaches. Section 4 proposes PrivAG mechanism for collecting the categorical-attributed graph. Section 5 designs an adaptive binning scheme to extend the privacy field to heterogeneous graph, and provides optimization techniques for extended PrivHG mechanism. Section 6 shows the extensive experimental results of PrivHG and baseline mechanisms. Section 7 reviews related literature. Finally, Section 8 concludes the paper.

## 2. LDP in Graph

In this section, two variants of local differential privacy in graph data are briefly introduced with their pros and cons. Then an eclectic notion is proposed to better trade-off privacy and utility for heterogeneous graph data in local settings.

Since its inception [6], differential privacy (DP) has become the standard for preserving private data. By introducing the concept of neighboring databases that only differ in one record, a randomized mechanism M under differential privacy constraint can guarantee statistical indistinguishability for these two databases *D* and $D^{\prime }$. Although differential privacy has been extensively developed, practical scenarios lead to new challenges in local settings, therefore local differential privacy (LDP) is proposed [8], which relies on no trustworthy data curator and protects individual privacy on local devices. The privacy definition in local settings is based on user’s perspective of local private data. As for graph data, two variants of LDP are given in [30] with different perspective, and we review two LDP definitions on local graph as follows:

**Definition** **1** (Node-based Local Differential Privacy [30])**.** *A randomized mechanism M satisfies ϵ-node local differential privacy if and only if for any two neighboring graph $G,G^{\prime }$ differing in one node, and any O∈range(M),*

$$Pr\left(M\left(G\right)\in O\right)\leq exp\left(\epsilon \right)\cdot Pr\left(M\left(G^{\prime }\right)\in O\right)$$

**Definition** **2** (Edge-based local Differential Privacy [30])**.** *A randomized mechanism M satisfies ϵ-node local differential privacy if and only if for any two neighboring graph $G,G^{\prime }$ differing in one edge, and any O∈range(M),*

$$Pr\left(M\left(G\right)\in O\right)\leq exp\left(\epsilon \right)\cdot Pr\left(M\left(G^{\prime }\right)\in O\right)$$

Despite the conventional privacy definitions of node-based LDP and edge-based LDP, there are certain drawbacks if applying them to heterogeneous graphs. On the one hand, node-based local differential privacy is a very promising and rigorous one, but directly applying node-based notion may introduce excessive noises and reduce the utility vastly. On the other hand, users may require a stronger notion than edge-based local differential privacy by protecting several equally sensitive attributed edges together, for the reason that similarly attributed relations deserve similar protection. Considering the privacy demand and the nature of attributed graph data, we combine the characteristics of these two notions, and propose an eclectic notion as attributewise local differential privacy(ALDP).

**Definition** **3** (Attributewise Local Differential Privacy)**.** *A randomized mechanism M satisfies ϵ-attributewise local differential privacy, if and only if for any two neighboring attributed local graph data $G,G^{\prime }$ differing in one attribute and related edges, and any O∈Range(M)*

$$Pr\left[M\left(G\right)\in O\right]\leq exp\left(\epsilon \right)\cdot Pr\left[M\left(G^{\prime }\right)\in O\right]$$

Through trading off the rigorousness of node-based LDP and utility of edge-based LDP, we define neighboring private data from attribute level, that is to say, two attributed local graphs are neighboring if one can be obtained from another by altering one certain attribute along with all related edges. Intuitively, the privacy budget ϵ in ALDP is split among a subset of edges, where ϵ in node-based LDP is split in all edges and ϵ in edgebased LDP is used as a whole. Both node-based LDP and edge-based LDP can be viewed as extreme cases of ALDP. In one extreme case, the whole graph has only one attribute, and altering it is equivalent to altering all the edges, then ALDP corresponds to nodebased LDP. In another extreme case, each edge of the whole graph has a distinct attribute value, and altering one certain attribute is equivalent to altering only one edge, then ALDP corresponds to edge-based LDP. Besides the extreme cases, ALDP in the nonextreme case actually trade-offs between the two definitions, thus achieving better estimation accuracy than the former and providing stronger privacy protection strength than the latter. In this paper, we aim to analyze edge-attributed graph under ALDP.

Some useful properties [8] of differential privacy provide theoretical guarantees for the design of subsequent mechanisms, the allocation of privacy budgets, and the optimization of perturbation results.

**Theorem** **1** (Sequential Composition [8])**.** *If randomized mechanism $M_{i}$ satisfies $\epsilon_{i}$-local differential privacy for i=1,…,k, then the sequential composition $M=(M_{1},\ldots ,M_{k})$ on private data G satisfies $\sum_{1}^{k}\epsilon_{i}$-local differential privacy.*

**Theorem** **2** (Parallel Composition [8])**.** *If randomized mechanism $M_{i}\left(G_{i}\right)$ satisfies $\epsilon_{i}$-local differential privacy for i=1,…,k, then the parallel composition $M=(M_{1}\left(G_{1}\right),\ldots ,M_{k}\left(G_{k}\right))$ on private data G satisfies $\max\epsilon_{i}$-local differential privacy.*

**Theorem** **3** (Postprocessing [8])**.** *If randomized mechanism M satisfies ϵ-local differential privacy, and f is a randomized mapping function, then f∘M satisfies ϵ-local differential privacy.*

## 3. Problem Definition and Naive Approach

### 3.1. Problem Definition

**Figure 1:**
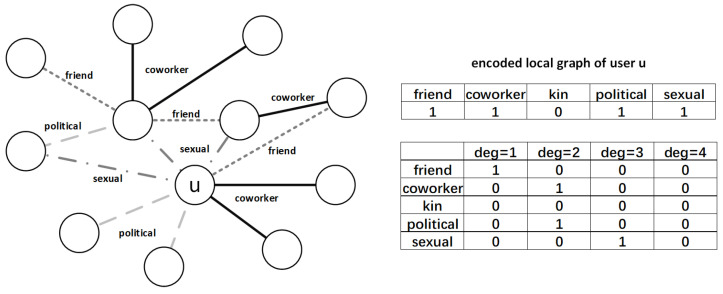
An example of attributed local graph $G^{u}$, right half is encoded vectors of $G^{u}$.

Consider an edge-attributed graph as an undirected graph G=(V,E,A), where *V* represents nodes in the graph, $E=\{e_{u,v}|u,v\in V\}$ represents edges, and each edge between two nodes is related to one attribute $a_{j}$ from the universal attribute set *A*. Without lose of simplicity, in this paper we assume that each local graph may have several attributes but each edge of the graph has only one attribute. A graph with multidimensional attributes is beyond the scope of this paper, and we leave it for our future work. We assume that there are totally |V| nodes, |E| edges and |A| attributes in graph *G*, which are all publicly known. Beyond the global parameters, local graph data $G^{i}$ is stored on each individual *i*’s device, and is considered as private. These private data include linked edges $E^{i}$ and possessed attributes $A^{i}$. Take Figure 1 as an example to encode local graph data, user *u* holds four attributes from the universal attribute set (friend, coworker, kin, political, sexual), so the attribute vector of *u* is represented as (1,1,0,1,1) with kinship as 0 as in upper right of Figure 1. There is one edge attributed as friend, which means the degree of attribute friend is 1, thus the first vector in lower right of Figure 1 is set to (1,0,0,0), so are other degree vectors. As for attributes not exist in graph, that rows are simply set to 0. The main notations are listed in Table 1.

The objective of this paper is to provide tools for data curators to analyze heterogeneous local graphs, while satisfying ϵ-local differential privacy. Precisely speaking, through collecting perturbed attribute vector and attribute-degree vectors, we focus on estimating two fundamental statistics:

- Attribute frequency estimation. The attribute frequency $\phi^{j}$ is the ratio of users who possessed certain attribute $a_{j}$ among whole users in the graph (e.g., fraction of users who installed certain social App among all Appstore users):
  (1) $$\begin{array}{c} \phi^{j}=\frac{\#\{G^{i}|\exists a_{j}\in A^{i}\}}{n} \end{array}$$
- Attribute-Degree distribution estimation. The attribute-degree distribution $\psi_{j}^{d}$ is attributed version of degree distribution. Formally speaking, $\psi_{j}^{d}$ is the number of nodes that have exactly *d* edges with attribute $a_{j}$:
  (2) $$\begin{array}{c} \psi_{j}^{d}=\frac{\#\{G^{i}|\exists a_{j}\in A^{i}\;and\;deg^{i}\left(a_{j}\right)=d\}}{n\cdot \phi^{j}} \end{array}$$

### 3.2. Data Preprocessing

Considering the practical flexibility, analyzing edge-attributed graph data still needs precaution before getting down to algorithm details. For attribute estimation, domain of real-world graph attributes can be enormous, and each user may possess several edge attributes in their local graph data. For simplicity, we assume, as in recent work [19,20], that the number of edge attributes in each user’s local graph is fixed by parameter *ℓ*. As for degree estimation, when user opting out one attribute under attributewise local differential privacy, related edges will also be altered simultaneously, thus brings the high sensitivity of graph analysis, which in the worst case may reach the maximum degree, |V|−1. Therefore, method to neutralize the effect of high sensitivity and retain better utility should be considered. Existing research studies mainly limit the magnitude of noises by projecting the original graph into a bounded graph with maximum degree equals θ.

In this paper, we first fix number of possessed attributes in each user’s local graph, i.e., $|A^{i}|=\ell$. If a user has more than *ℓ* edge attributes in her local graph, she randomly sample *ℓ* attributes from the origin graph, together with related edges, forming a new graph with fixed *ℓ* edge attributes. For user with less than *ℓ* attributes, $\ell -|A^{i}|$ dummy items are padded to her graph, which are ignored by data curator in analyzing process. Then, for each attribute $a_{j}$ possessed in user’s local graph $G_{i}$, we set the maximum number of related edges in local graph as θ. When the number of edges with attribute $a_{j}$ exceeds the given parameter, we truncate extra edges and bound the degree with θ. After the preprocessing of padding and truncating, we get the resulting attributed local graph ${\bar{G}}^{i}$. With the bounded local graph, we can further compare the allocation of privacy budget among different privacy notions: for attribute frequency estimation, budget $\epsilon_{a}$ in ALDP and edge LDP is used as a whole, while it is split as $\epsilon_{a}/\ell$ for each attribute in node LDP; for degree estimation, budget $\epsilon_{d}$ in edge LDP is used as a whole, while it is split as $\epsilon_{d}/\theta$ for each edge in ALDP and node LDP. In summary, ALDP strike a balance between edge LDP and node LDP.

### 3.3. Naive Approach

The first intuitive approach is adopting Laplace mechanism under ALDP, each user preprocesses her local graph into a bounded one, calculates numerical statistics and perturbs the statistics with Laplace noises. Specifically, given the initial parameters (including attribute set size *ℓ*, maximum degree θ and privacy budget ϵ), each user first encodes the bounded graph as a numerical vector, in which each bit representing correlated number of edges with certain attribute, then adds Laplace noises sampled from $Lap(\frac{\Delta }{\epsilon })$. In the bounded local graph, changing one attribute will change the numerical vector at most 2θ+1 bits, thus the sensitivity bound is Δ=2θ+1. Based on the Laplace approach, attribute-degree distribution can be estimated by aggregating the perturbed vectors from all users, and attribute frequency estimation can be derived from attributes with nonzero degree. However, the error of estimation is related to θ and the results can be highly inaccurate with a large θ, and when estimating attribute frequency solely, the sensitivity should be 2 instead of 2θ+1, thus extra noises are added to the origin data.

Another naive approach to solve the problem is applying Randomized Response [33], and separately perturbing attribute possession and degree distribution by flipping two different coins. In particular, given the initial parameters, each user first encodes local graph as an attribute vector $\phi^{a}$ and degree vectors $\psi^{d}$, where $\phi^{a}$ is a binary vector indicating local attribute possession and $\psi^{d}$ consists of *m* one-hot vectors denoting the degrees of corresponding attribute. By preprocessing and encoding local graphs, each user splits privacy budget ϵ in two parts $\epsilon_{1}$ and $\epsilon_{2}$ to perturb attribute and degree vectors respectively. In the attribute perturbation phase, the user splits $\epsilon_{1}$ into 2ℓ parts and invokes GRR [11], which is an enhanced version of Randomized Response, to perturb bits in attribute vector with flipping probability: $p=\frac{1}{exp(\frac{\epsilon_{1}}{2\ell })+1}$. In the degree perturbation phase, the user splits $\epsilon_{2}$ into 2θ parts, and perturbs the bits in degree vectors with probability: $p=\frac{1}{exp(\frac{\epsilon_{2}}{2\theta })+1}$. After the local perturbation, data curator collects perturbed vectors from all users and performs an unbiased estimation of attribute frequency and attribute-degree distribution. We regard this GRR approach as a baseline to our problem.

By observing these two approaches, some hurdles can be found. The conventional Laplacian mechanism is easy to implement, however, the noises added to origin data is θ-related, and the choice of θ is empirical and relies on specific graph data. Invoking Randomized Response twice as in GRR approach is a remedy to the problem, but pays the price of utility degrading by splitting privacy budget too fractionally. Furthermore, the attribute frequency and its degree distribution in one local graph should be correlated, and these two naive approaches fail to capture this property. In the next chapter, we tackle these hurdles in our PrivAG mechanism.

## 4. PrivAG Mechanism

### 4.1. General Mechanism

In order to tackle the aforementioned shortage of intuitive approach, we manage to reduce the fragmentation of privacy budget and retain the correlation between attribute possession and degree in PrivAG. The main idea of the mechanism is to first output an randomized attribute subset of fixed size *k*, where *k* relies on given parameters, and then accordingly perturb degree vectors based on the result of randomized attribute data. Specifically, PrivAG is comprised of two components, randomization and estimation component:

Randomization component. This component includes two phases that separately randomize attribute and degree vectors. One previously observed hurdle of naive approach is tiny split privacy budget and excessive noises, and the key coping idea for attribute randomization, which is inspired by the recent work [19], is to locally sample an attribute subset of size *k* as a whole to reduce introduced noises, without splitting privacy budget $\epsilon_{1}$. As for degree randomization, inspired by research studies [34,35], we take OUE [34] as building block for degree randomization in this paper, which eliminates the effect of θ on the variance and transmits bit 1s and 0s differently. Note that the OUE method is replaceable in our mechanism, and GRR [33] or other methods could be an alternative for extremely sparse graphs with $\theta <3exp(\epsilon_{2})+2$. After the randomization, additional postprocessing is executed to sustain the correlation between attribute and degree. The algorithmic detail of randomization component is presented in next subsection.

Estimation component. To reduce computational cost, data curator first broadcasts needed parameters to every user in the initializing phase of PrivAG, including public parameters and set size *k* in randomization component. *k* is calculated based on other public parameters, and the optimal $k^{*}$ can be derived to further maximize utility, through treading off the theoretical error bounds of attribute frequency and degree distribution estimation. After each user invokes randomization component, data curator collects the perturbed data, and make an unbiased estimation about attribute and degree. Next, we present the two components in detail.

### 4.2. Data Randomization

Attribute randomization. In this phase, each user *i* encodes attribute set ${\bar{A}}^{i}\subseteq {\bar{G}}^{i}$ as a binary vector $v_{a}^{i}=\left(v_{a_{1}}^{i},\ldots ,v_{a_{m}}^{i}\right)$, then samples and outputs *k* elements from $v_{a}^{i}$ with noisy probability consuming privacy budget $\epsilon_{1}$. Denote the output as ${\tilde{v}}_{k}^{i}=\left({\tilde{v}}_{a_{1}}^{i},\ldots ,{\tilde{v}}_{a_{k}}^{i}\right)$, which is one possible result from output domain $v_{k}$ of all *k*-sized attribute vectors. This randomization phase is implemented based on the general Exponential Mechanism, which outputs element of maximum utility score *u* with the probability proportional to $exp(\frac{\epsilon u}{2\Delta u})$.

Given an input $v_{a}^{i}$ and output domain $v_{k}$ of all *k*-sized attribute vectors, we first define the essential utility function $u(v_{a}^{i},{\tilde{v}}_{k}^{i})$ to score the similarity between *m*-sized vector $v_{a}^{i}$ and *k*-sized vector ${\tilde{v}}_{k}^{i}$ pairs. To keep the noisy probabilities stable, we define our utility function as a indicator function, indicating whether the $\ell_{1}$ distance on the sampled *k* elements between $v_{a}^{i}$ and ${\tilde{v}}_{k}^{i}$ is within *k*:

$$u\left(v_{a}^{i},{\tilde{v}}_{k}^{i}\right)=\left[\right|v_{a}^{i}-{\tilde{v}}_{k}^{i}{|}_{1}<k]$$

It can be derived that the sensitivity of utility function is 1:

$$\begin{array}{cc} \Delta u & =\max_{{\tilde{v}}_{k}^{i}\subseteq v_{k}}\max_{\left|\right|v_{a}^{i}-v_{a}^{i^{\prime }}{\left|\right|}_{1}\leq 1}\left|u\left(v_{a}^{i},{\tilde{v}}_{k}^{i}\right)-u\left(v_{a}^{i^{\prime }},{\tilde{v}}_{k}^{i}\right)\right|\\ & =\max_{{\tilde{v}}_{k}^{i}\subseteq v_{k}}\max_{\left|\right|v_{a}^{i}-v_{a}^{i^{\prime }}{\left|\right|}_{1}\leq 1}\left|[\right|v_{a}^{i}-{\tilde{v}}_{k}^{i}{|}_{1}<k]-[|v_{a}^{i^{\prime }}-{\tilde{v}}_{k}^{i}{|}_{1}<k]|=1 \end{array}$$

Through defining the low-sensitivity utility function $u(v_{a}^{i},{\tilde{v}}_{k}^{i})$, the noisy probability of outputting ${\tilde{v}}_{k}^{i}$ with input $v_{a}^{i}$ is given by:

$$Pr\left[M\left(v_{a}^{i}\right)={\tilde{v}}_{k}^{i}\right]\propto exp\left(\frac{\epsilon_{1}u\left(v_{a}^{i},{\tilde{v}}_{k}^{i}\right)}{\Delta u}\right),{\tilde{v}}_{k}^{i}\subseteq v_{k}$$

Substituting Δu=1, the attribute randomization probability can be derived by aggregating all the proportional probabilities above:

$$Pr\left[M\left(v_{a}^{i}\right)={\tilde{v}}_{k}^{i}\right]=\frac{exp(\epsilon_{1}u\left(v_{a}^{i},{\tilde{v}}_{k}^{i}\right))}{\sum_{{\tilde{v}}_{k}\in v_{k}}exp\left(\epsilon_{1}u\left(v_{a}^{i},{\tilde{v}}_{k}\right)\right)}$$

The implementation of attribute randomization phase is illustrated in the Algorithm 1 from line 2 to line 14. During the line 2 and line 8, each user computes a series of probabilities, where Σ is the normalizer $\Sigma =\sum_{{\tilde{v}}_{k}\in v_{k}}exp\left(\epsilon_{1}u\left(v_{a}^{i},{\tilde{v}}_{k}\right)\right)$, and each $p_{i}$ with i∈[0,k] represents the probability that the number of selected origin attributes $a\in {\bar{A}}^{i}$ is exactly *i*, $p_{i}=Pr\left[\#\left\{a_{j}|a_{j}\in A^{i}\;and\;{\tilde{v}}_{k}\left[a_{j}\right]=1\right\}=i\right]$. Since *u* is an indicator function, the output domain of $v_{k}$ contain m+ℓk outputs, and u=0 when selecting *k* attributes from noninitial m+ℓ−ℓ=m attributes, so Σ can be calculated with given parameters. The probabilities $p_{i}$ is calculated iteratively. From line 8 to line 14, each user randomly generates a number $k_{s}$ based on the previous probabilities, separately samples $k_{s}$ attributes from ${\bar{A}}_{i}$ and samples $k-k_{s}$ attributes from the rest noninitial attributes set, and vectorization the union set as ${\tilde{v}}_{k}^{i}$, which is the perturbed attribute vector to be contributed.
**Algorithm 1** Data Randomization Component (DRC).**Input:** attributed local graph $G^{i}$, privacy budget ϵ, *m*, *l*, θ
**Output:** perturbed attribute–degree vectors $\hat{s}\in S$
   1: //locally truncate and pad origin graph
   2: ${\bar{G}}^{i}\leftarrow pre-processing\left(G^{i}\right)$
   3: //attribute perturbation
   4: Σ←mk+exp(ϵ1)(m+ℓk−mk)
   5: p0←mkΣ
   6: **for**
i∈[1,k]
**do**
   7:     pi←pi−1+exp(ϵ1)miℓk−iΣ
   8: **end for**
   9: $r_{att}\leftarrow random\left(0.0,1.0\right)$
   10: $k_{s}\leftarrow 0$
   11: **while**
$p_{k_{s}}\leq r_{att}$
**do**
   12:     $k_{s}\leftarrow k_{s}+1$
   13: **end while**
   14: ${\tilde{v}}_{k}\leftarrow vectorization\left(sample\left(k_{s},A^{i}\right)\cup sample\left(k-k_{s},A-A^{i}\right)\right)$
   15: **for**
$a_{j}\in {\tilde{v}}_{k}\;and\;a_{j}\notin G^{i}$
**do**
   16:     t←random(1,θ)
   17:     $u_{t}^{j}\leftarrow 1$
   18: **end for**
   19: //degree randomization
   20: **for**
$a_{j}\in {\tilde{v}}_{k}$
**do**
   21:     **for** t∈[1,θ] **do**
   22:         Perturbs as
$$Pr\left[{\tilde{u}}_{t}^{j}=1\right]=\left\{\begin{array}{cc} p_{d}=\frac{1}{2}, & u_{t}^{j}=1\\ q_{d}=\frac{1}{e^{\epsilon_{2}/k}+1}, & u_{t}^{j}=0 \end{array}\right.$$
   23:     **end for**
   24: **end for**
   25: **for**
$a_{j}\in A$
**do**
   26:     ${\tilde{u}}^{j}\leftarrow {\tilde{u}}^{j}a_{j}$
   27: **end for**
   28: **return**
${\tilde{v}}_{k}$ and ${\tilde{u}}_{d}$


Degree randomization. As mentioned above, we adopt OUE to serve as our attributedegree perturbation primitive. To be specific, for a individual’s local graph $G^{i}$, after projected to ${\bar{G}}^{i}$, the number of edges with every attribute is known and limited, thus can be encoded as one-hot attribute-degree vector $u^{j}=\left[u_{0}^{j},\ldots ,u_{\theta }^{j}\right]$, where the subscript *j* stands for attribute $a_{j}\in A^{i}$ and only $deg^{i}\left(a_{j}\right)$-th bit is 1 in this degree vector of attribute $a_{j}$. For one-hot vectors like $u_{d}$, OUE takes noncomplementary probabilities for bits 1 and 0, bit 1 in $u_{d}$ stays as 1 in ${\tilde{u}}_{d}$ with probability p=1/2, in the meantime, bits 0 in $u_{d}$ are flipped with probability *q*. The general randomization process can be sketched as:

$$Pr\left[M\left({\tilde{u}}_{i}=1\right)\right]=\left\{\begin{array}{cc} p, & u_{i}=1\\ q, & u_{i}=0 \end{array}\right.$$

One shortage of baseline approach is that it fails to capture the intrinsic correlation between attribute and its degree, for example when $a_{j}$ is perturbed as 0 after attribute randomization phase, the related degree vector $u_{d}^{j}$ should also be 0 after perturbation. After the attribute randomization phase in PrivAG, there are *k* selected attributes as a whole to be perturbed as 1, with the rest bits in attribute vector $v_{a}$ as 0, thus there should be *k* related vectors $u_{d}$ with nonzero degree. Degree randomization process is executed *k* times for each selected attribute in ${\tilde{v}}_{k}^{i}$, with split privacy budget $\epsilon_{2}/k$, and for the rest attributes not in ${\tilde{v}}_{k}^{i}$, the degree vector is postprocessed to stay 0 with attributes simultaneously. Furthermore, parameter *k* take a role in both phases, and the optimal *k* is determined by estimation of both phases in theoretical analysis section. Therefore, the correlation between attribute and degree is retained. As shown from line 18 to line 24, in degree randomization phase, each user splits privacy budget as $\epsilon_{2}/k$, and utilizes each share to flip one attribute-degree vector of selected attribute from attribute randomization phase.

By combining the two phases, the Data Randomization Component(DRC) is presented in Algorithm 1, in the initializing stage, each user gets public parameters from data curator, preprocesses local graphs, and divides privacy budget as $\epsilon =\epsilon_{1}+\epsilon_{2}$ for subsequent randomization. In the final stage, each user multiples the perturbed degree vectors with the related attribute vector value, ensuring that the two phases are perturbed simultaneously.

### 4.3. Distribution Estimation

In this subsection, we present the complete PrivAG framework, including attribute frequency and attribute-degree distribution estimation component. In Algorithm 2, each user executes DRC on her edge-attributed local graph, and contributes the sanitized results to data curator. After collecting the perturbed data from all users, data curator aggregates the results and accordingly infers the attribute frequency $\phi^{a}$ and attribute-degree distribution $\psi^{d}$. The thorough estimation phase of PrivAG framework is given below.
**Algorithm 2** PrivAG.**Input:** local graphs *G*, privacy budget ϵ
**Output:** attribute frequency $\phi^{a}$, attribute–degree distribution $\psi^{d}$
   1: //user-side randomization
   2: each user locally perturbs $G^{i}$ by DRC, and report ${\tilde{v}}_{k}^{i}$ and ${\tilde{u}}_{d}^{i}$
   3: //count bits 1 in the randomized vectors
   4: $c_{j}\leftarrow count\left({\tilde{v}}_{k}\right)$
   5: $d_{t}\left(a_{j}\right)\leftarrow count\left({\tilde{u}}_{d}\right)$
   6: //estimate from recorded counts
   7: **for**
j∈[1,m]
**do**
   8:     $\phi^{j}=\frac{c_{j}/n-q_{a}}{p_{a}-q_{a}}$
   9: **end for**
   10: **for**
j∈[1,m]andt∈[0,θ]
**do**
   11:     $\psi_{j}^{t}=\frac{d_{t}\left(a_{j}\right)/n-q_{d}}{p_{d}-q_{d}}$
   12: **end for**
   13: **return**
$\phi^{a}$ and $\psi^{d}$


Attribute frequency. In this phase, data curator aggregates bit 1s in perturbed vector ${\tilde{v}}_{k}$ from *n* individuals as a counting vector $c_{j}=\#\left\{{\tilde{v}}_{k}^{i}|{\tilde{v}}_{k}^{i}\left[a_{j}\right]=1\right\}$ and calibrates the frequency. During the calibration process, two probabilities are critical, which we denote as $p_{a}$ and $q_{a}$. For a user *i* and an attribute $a_{j}\in A$, if $a_{j}$ both appears in the origin vector of $A^{i}$ and the perturbed result ${\tilde{v}}_{k}$, the probability is denoted as $p_{a}$:

(3) pa=Pr[v˜k[j]=1|vj=1]=exp(ϵ1)m+ℓ−1k−1mk+exp(ϵ1)(m+ℓk−mk)

Similarly, if $a_{j}$ is beyond user’s possessed attributes $a_{j}\notin A^{i}$, but the perturbed result ${\tilde{v}}_{k}$ contains $a_{j}$, then the probability is denoted as $q_{a}$:

(4) qa=Pr[v˜k[j]=1|vj=0]=m−1k−1+exp(ϵ1)(m+ℓ−1k−1−m−1k−1)mk+exp(ϵ1)(m+ℓk−mk)

By calculating these probabilities $p_{a},q_{a}$ and the counting vector $c_{a}$, the unbiased estimation of attribute frequency $a_{j}$ is:

(5) $$\begin{array}{c} \phi^{j}=\frac{c_{j}/n-q_{a}}{p_{a}-q_{a}} \end{array}$$

Attribute-Degree distribution. The estimation of attribute-degree distribution is pretty similar to the previous phase. Data curator first aggregates bits 1s in vectors ${\tilde{u}}_{d}$ contributed by *n* individuals as a counting vector $d_{t}\left(a_{j}\right)=\#\left\{{\tilde{u}}_{d}^{i}|{\tilde{u}}_{t}^{i}\left[a_{j}\right]=1\right\}$. By combining the two important probabilities already given in Algorithm 1: $p_{d}=\frac{1}{2}$ and $q_{d}=\frac{1}{1+e^{\epsilon_{2}/k}}$. Then data curator estimates the attribute-degree distribution as:

(6) $$\begin{array}{c} \psi_{j}^{t}=\frac{d_{t}\left(a_{j}\right)/n_{j}-q_{d}}{p_{d}-q_{d}} \end{array}$$

With split privacy budget $\epsilon =\epsilon_{1}+\epsilon_{2}$, the above mechanism PrivAG satisfies ϵ-attributewise local differential privacy. Upon analysis, the categorical-attributed graph with PrivAG mainly has two kinds of errors, relating to two estimation objectives. Next, we theoretically analyze these two errors and optimize key parameter to reduce estimation variance.

Error analysis on attribute frequency estimation. Based on the probabilities calculated in the previous subsection, the variance of an attribute $a_{j}\in A$ frequency is:

(7) $$Var\left[\phi^{j}\right]=\frac{nq_{a}\left(1-q_{a}\right)}{{\left(p_{a}-q_{a}\right)}^{2}}$$

Error analysis on attribute-degree distribution estimation. Similarly, the variance of an attribute $a_{j}\in A$ frequency is:

(8) $$Var\left[\psi_{j}^{t}\right]=\frac{nq_{d}\left(1-q_{d}\right)}{{\left(p_{d}-q_{d}\right)}^{2}}$$

## 5. PrivHG: Extending to Heterogeneous Graph

The aforementioned PrivAG mechanism is efficient and effective to perform analysis tasks for categorical-attributed graph. In this section, the privacy field is generalized from categorical attribute to heterogeneous attributes, such as the heterogeneous social networks, social–financial networks and geosocial networks, and an enhanced version of PrivAG mechanism (denoted as PrivHG) is presented to aggregate two statistics $\phi^{a}$ and $\psi^{d}$ of local heterogeneous graph. The estimation accuracy and computation overhead of PrivHG are further optimized both in user-side randomization component and server-side estimation component.

The premise of extending PrivAG from categorical-attributed graph to heterogeneous graph is to collect categorical and numerical attribute possessions #a|a∈A and mixedattributed edge degrees {deg(a)|a∈A}. An available approach is to separately collect these mixed statistics: leave categorical-attributed data to PrivAG and aggregate numericalattributed data with hierarchy-based approach. The hierarchy approach commonly constructs an additional hierarchical structure and perturbs private data with multiple privacy granularity. Despite the additional computation cost of the hierarchical data structure building process, the limited privacy budget ϵ will be allocated proportionately between PrivAG and hierarchy-based mechanisms when separately randomizing categorical-attributed and numerical-attributed data, which is also inefficient and impractical. Therefore, it is inappropriate to apply hierarchy-based approach for numerical data of heterogeneous graph in PrivHG mechanism. Another way is to apply binning-based approach to segment continuous numerical attributes $a\in A_{n}$ into discrete *r* intervals with binning scheme $B=(b_{1},b_{2},\ldots ,b_{r})$, and deal with them equally as categorical data. By applying binningbased approach in PrivHG mechanism, categorical-attributed and numerical-attributed statistics $a|a\in A_{c}\cup B$ and $\left\{deg\left(a\right)|a\in A_{c}\cup B\right\}$ can be aggregated under the same process, and privacy budget ϵ is utilized as a whole. In the following, PrivHG adopts binning-based approach as a building block to collaboratively analyze private heterogeneous graph along with PrivAG, a resizing binning technique is further designed in PrivHG to handle the large domain problem of heterogeneous graph, which reduces the aggregation and estimation error compared with straightforward application of binning-based approach.

Despite the intuitive outline of the extended PrivHG mechanism, there are still limitations on the details of aggregating heterogeneous graph data, leaving room for following improvement.

- In the initialization process of PrivHG mechanism, the binning scheme $B=(b_{1},b_{2},\ldots ,b_{r})$ is directly applied to the local data to aggregate statistics, thus determines the estimation accuracy brought by subsequent truncation and perturbation processes. Since the heterogeneous graph data are potentially distributed unevenly but truncated uniformly with maximum degree parameter θ, different binning scheme B∈B, which groups data with different granularity of sparsity, affects the gap between truncation range [1,θ] and actual data range $\left[min\left(deg\left(b_{i}\right)\right),max\left(deg\left(b_{i}\right)\right)\right]$ for each bin $b_{i}\in B$, therefore having an impact on the accuracy of subsequent attribute-bins-related estimation for $a_{j}\in b_{i}$. To be more specific, considering two extreme cases: if the binning scheme *B* is too fine, most bins $b_{i}\in B$ contain only sparse attributed edges and aggregation of these sparse data falls far below the truncation threshold $max(deg\left(b_{i}\right))\ll \theta$, then excessive θ-related noises are introduced into these sparse bins and associated attributed graph during perturbation process; On the other hand, a too coarse binning scheme *B* groups numerous attributed edges together, then the aggregated statistics of these large bins $b_{i}\in B$ may exceed the truncation parameter θ too much $max(deg\left(b_{i}\right))\gg \theta$, resulting in enormous error due to excessive attributed edges being truncated. Note that this unevenly distributed but uniformly truncated limitation applies for both categorical-attributed and numericalattributed data in heterogeneous graphs. Therefore, finding optimal binning scheme in unified PrivHG mechanism is critical, and perturbation with inappropriate binning scheme could suffer from high randomization error with sparse data and high truncation error with dense data.
- During the randomization process of heterogeneous graph, the intrinsic correlations between attributed edges need to be reflected in the simultaneous randomization of attributes (bins) and degrees, and retained in the estimation of attribute frequency $\phi^{a}$ and degree distribution $\psi^{d}$. Especially, if a nonpossessed attribute $\left(a_{j}=0\right)$ is perturbed as a possessed one on $\left(a_{j}=1\right)$ when perturbing a private local graph, a fake attributed degree $deg(a_{j})$ needs to be generated as a counterpart; On the contrary, if a possessed attribute $\left(a_{j}=1\right)$ is perturbed as a nonpossessed one on $\left(a_{j}=0\right)$, related degree $deg(a_{j})$ is set to 0. The fake degree $deg(a_{j})$ in PrivAG mechanism is randomly generated from range [1,θ] without prior knowledge, which skews the estimation results of degree distribution $\psi^{d}$.
- The sampling size *k* of randomized data set is determined on the server side without considering local devices’ capabilities, which lead to O(k∗θ) computation and communication overhead on the user side. A large *k* represents that lots of data needs to be sampled, randomized and contributed from each user, which means much burden to user’s device. However, practical local devices have various capabilities, and imposing heavy burden to the low-capability local devices in turn brings difficulties to data collection.
- The randomization strength and estimation accuracy in PrivAG mechanism is controlled by the privacy budget ϵ. When ϵ is split many times among heterogeneous graphs, the outliers generated by data randomization component may obscure graph data characteristic and have a relatively huge impact on the estimation results, but PrivAG mechanism is lack of corresponding techniques to correct randomization outliers and neutralize estimation variance.

**Figure 2:**
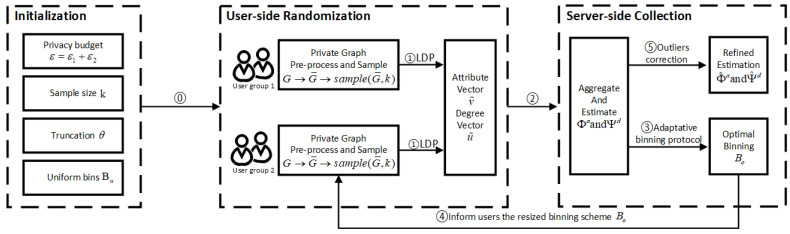
Overview of PrivHG mechanism.

The overview of PrivHG mechanism is shown in Figure 2, which mainly extends the privacy field to heterogeneous graph data and optimizes the above limitations. Taking the real-world applications of heterogeneous social network and social–financial network as examples, the brief process of running PrivHG mechanism can be summarized as: First, during the initialization phase, the whole heterogeneous social graph or social-financial network is divided as two user groups. Users in group 1 preprocess the numerical attributes (e.g., contacting time intervals in heterogeneous social network or fund transfer amount in social–financial network) according to the binning scheme, and encode their local graph data as illustrated in Figure 1. Then the numerical-attributed data (e.g., contacting time intervals and fund transfer amount) and categorical-attributed data (e.g., social linkage type and financial activity type) are equally randomized and collected with randomization mechanism. After the data curator aggregates the statistics, a generalized optimal binning scheme is output, covering mixed attributes with minimal estimation error. In the following phase, the optimal binning scheme and necessary parameters are informed to user group 2, and each user preprocesses and randomizes his/her local data with optimization techniques. Finally, these randomized vectors are aggregated by the server, then unbiased estimations about the data distribution of heterogeneous social network or social–financial network are generated. Specifically, the techniques of extending to PrivHG mainly include the following components: Adaptive Binning Scheme is firstly proposed to find an optimal binning scheme $B_{o}\in B$ for mixed attributes based on a portion of the heterogeneous graph data (Section 5.1). The binning $B_{o}$ in ABS strikes a balance between truncation and perturbation error, ensuring that the final aggregated statistics are approximately around the threshold $max(deg\left(b_{i}\right))\approx \theta$ for $b_{i}\in B_{o}$. Then, the byproducts of Adaptive Binning Scheme enable subsequent optimizations. During the perturbation process, the sample set size *k* is chosen by trading off communication overhead and estimation accuracy (Section 5.2), and correlated fake degrees are calibrated based on the estimated data distribution in ABS rather than random values (Section 5.2). Finally, considering the heterogeneous graph data properties, the aggregated statistics are corrected by filtering out the outliers (Section 5.3).

### 5.1. Adaptive Binning Scheme

This subsection elucidates the process of finding the optimal binning scheme $B_{o}$ in Figure 2. As previously stated, binning schemes are designed to discretize numericalattributed data, so that heterogeneous graphs can be perturbed uniformly with PrivHG mechanism. As different binning schemes influence the final estimation accuracy differently, the intuition to find a proper binning scheme requires keeping aggregated statistic of each bin as close to the truncation threshold as possible $max(deg\left(b_{i}\right))\approx \theta$ for $b_{i}\in B_{o}$, in the mean time minimizing both the estimation error from perturbations and the truncation error from binning, and reducing the dependence on background knowledge of data distribution.

Two basic binning schemes for heterogeneous graph data are uniform binning and geometric binning. Uniform binning is pretty straightforward and intuitive. For numerical attribute range a∈[1,w], uniform binning divides it into bins with equal width, $B=b_{i}|i\in \left(1,r\right)$ where $b_{i}=\left(1+\left(i-1\right)*\delta ,1+i*\delta \right)$ and $\delta =\frac{w-1}{r}$. Geometric binning is another feasible scheme, where the bins are covered by a geometric series $\delta^{i}$ and the width of bins varies from narrow to wide, which mimics the long tail distribution nature of some graph data. Formally speaking, the [1,w] interval is divided geometrically as $b_{i}=\left(1+\delta^{i-1},1+\delta^{i}\right)$, where i∈(1,r) and δ is a predefined parameter controlling the variations of bin width. However, there are drawbacks when applying the two basic binning schemes. First of all, finding the parameter δ that controls the width of bins in the binning schemes requires practical experience, and to guarantee finding the optimal parameter is a nontrivial effort. Second, the two binning schemes rely on certain data distribution to achieve accurate estimation and they may perform poorly in other scenarios, for example, uniform binning suffers from the unevenly distributed but uniformly truncated problem, and geometric binning suffers from nongeometric data distributions. Third, once the two binning schemes are defined, they are only suitable for the covered graph and not applicable to other graphs. Last but not least, uniform binning scheme may cover a set of categorical attributes and geometric binning scheme may cover a set of numerical attributes, but the PrivHG mechanism requires a unified scheme to cope with mixed attributes of heterogeneous graphs. As a precursor subtask of the PrivHG mechanism, we propose Adaptive Binning Scheme (ABS), which integrates the merits of above schemes and allows PrivHG to be conveniently extended to the mixed-attributed data of heterogeneous graph.

As shown in Algorithm 3, ABS first divides numerical attribute as discrete intervals with basic binning scheme (we take uniform binning $B_{u}=\left(1+(i-1)*\delta ,1+i*\delta \right)$ where i∈(1,r) and $\delta =\frac{b-1}{r}$ in PrivHG for simplicity, while geometric or other binning is alternative), and aggregates both numerical-attributed and categorical-attributed data of heterogeneous graph. Then ABS estimates the error and cost variations of resizing and merging the bins for all possible binning schemes B∈B, and finds the binning scheme with minimum overall cost. Finally the optimal binning $B_{o}$ is distributed to subsequent subtasks. Comparing with uniform and geometric binning scheme, the benefits of ABS are evident: 1. The large domain problem of heterogeneous graph and predefined binning is neutralized in ABS by combining sparse bins and reducing overall bin counts. 2. Attributed data that are comparatively below the maximum degree threshold θ are collected simultaneously, trading off truncation error and perturbation noises. 3. ABS is feasible for both numerical- and categorical-attributed graph data, which collects heterogeneous data under one mechanism and avoids overdivision of privacy budget. 4. Byproduct of ABS provides access to subsequent optimization techniques of PrivHG, which is illustrated in the following subsection.

Based on the essential objective of adaptive binning scheme is to ensure that the maximum aggregated degree of each bin is as close to the truncation parameter θ as possible, while ensuring the overall cost of executing ABS as small as possible. We formalize the ABS objective as minimizing the following three components of overall cost under the constraint of predefined parameter θ:

Binning Resize Cost. This component captures the cost of binning and truncating processes for the resulting estimation, which mainly introduced by the resizing from basic bins to optimal bins. For aforementioned basic bins with fixed bin size for single attribute a∈A, if the correlated maximum degree is below the truncation threshold max(deg(a))<θ, then vacant bits $\left[u_{max(deg(a))+1},\ldots ,u_{\theta }\right]$ in the encoded degree vector *u* are randomized as outliers after executing the perturbation mechanism with probability $q_{d}=\frac{1}{1+e^{\epsilon_{2}/k}}$, which further reduces the estimation accuracy. The larger deviation between correlated maximum degree and parameter |θ−max(deg(a))|, the more vacant bits are randomized as outliers, therefore the higher estimation error is. By merging sparse and lowdegree attributed data (basic bins), binning scheme *B* enables that the aggregated maximum degree of merged attributes/bins (b∈B) is close to truncation threshold max(deg(b))≈θ, which reduces the error of perturbing the vacant bits in the related degree vectors. Due to the reduction of vacant bits, binning part of overall resizing cost in general is a negative value. However, under extreme circumstances, some merged bins may also lead to extra data being truncated. For the merged attributes/bins a∈b, if the related degree exceed θ, then extra truncating cost is denoted by degrees $\sum_{a\in b}deg\left(a\right)-theta$. On the other hand, if the maximum aggregated degree of merged bin max(deg(b)) is still below the truncating parameter θ, then additional truncating cost of resizing this bin is 0. Summing the binning costs and truncating costs up, the overall resizing cost is given as below, where *B* is a binning scheme, $q_{d}$ is the probability of randomizing vacant bits as 1 and $\epsilon_{2}$ is privacy budget for degree randomization.

(9) $$\begin{array}{cc} RC(B,u_{d},\theta ,\epsilon_{2},k) & =BC\left(B,u_{d},\theta ,\epsilon_{2},k\right)+TC\left(B,u_{d},\theta \right)\\ \; & =\sum_{b_{i}\in B}(|\theta -max\left(deg\left(b_{i}\right)\right)|q_{d}-\sum_{a\in b_{i}}|\theta -max\left(deg\left(a\right)\right)\left|q_{d}\right)\\ \; & +\sum_{b_{i}\in B}max\left(\sum_{a\in b_{i}}deg\left(a\right)-\theta ,0\right)\\ \; & =\sum_{b_{i}\in B}(\frac{1}{1+exp(\epsilon_{2}/k)}(|\theta -max\left(deg\left(b_{i}\right)\right)|-\sum_{a\in b_{i}}|\theta -max\left(deg\left(a\right)\right)\left|\right)\\ \; & +max(\sum_{a\in b_{i}}deg\left(a\right)-\theta ,0)) \end{array}$$

Attribute Randomization Cost. This component captures the cost brought by binning schemes for the attribute randomization. After ABS merging bins with attribute a∈A (or basic bin b∈B) on the server side, the possession of local attributes is replaced by the possession of local merged bins, thus ${\bar{v}}_{i}=1$ if $\exists v_{a}=1$ and $a\in b_{i}$, and ${\bar{v}}_{i}=0$ if $\forall v_{a}=0$ and $a\in b_{i}$. During local randomization component, if a merged bin $b_{i}\in B$ is possessed by user, the indicating bit in binning vector is set to 1 ${\bar{v}}_{i}=1$, which is equivalent to all corresponding attribute bits being estimated as 1 $v_{a}=1$ for $a\in b_{i}$, while these attributes may not all be possessed by local user and the actual value of these bits may be 0. Attribute randomization cost comes from the difference between indicating bits in resized binning vector $\left({\bar{v}}_{1},\ldots ,{\bar{v}}_{i},\ldots ,{\bar{v}}_{r}\right)$ for merged bins $b_{i}\in B$ and indicating bits in attribute vector $\left(v_{1},\ldots ,v_{j},\ldots ,v_{m}\right)$ for attributes $a_{j}\in A$, which is formalized as $AC(B,{\bar{v}}_{b},v_{a})$.

(10) $$AC\left(B,{\bar{v}}_{b},v_{a}\right)=\sum_{b_{i}\in B}\sum_{a_{j}\in b_{i}}\left|{\bar{v}}_{i}-v_{j}\right|=\sum_{b_{i}\in B}{\bar{v}}_{i}\left(\right|b_{i}\left|-\sum_{a_{j}\in b_{i}}v_{j}\right)$$

Degree Estimation Cost. This component captures the cost brought by binning scheme for the degree estimation. When aggregating attributed degrees based on the binning scheme *B* in ABS, the degree of each attribute *a* is estimated as the average degree of related bin $b_{i}\in B$ for $a\in b_{i}$. Furthermore, if the merged degrees of bins exceed truncation parameter θ, extra edges are truncated on local devices, then the aggregated degree of each bin $b_{i}\in B$ is the minimum value of parameter θ and sum of attributed degree $deg\left(b_{i}\right)=\sum_{a\in b_{i}}deg\left(a\right)$; therefore, the estimated degrees of the including attributes are replaced by the statistical average $\hat{deg}\left(a\right)=\frac{deg(b_{i})}{|b_{i}|}$ for $a\in b_{i}$. Degree Estimation Cost comes from this deviation.

(11) $$\begin{array}{cc} DC\left(B,u_{d},\theta \right)=\sum_{b_{i}\in B}\sum_{a\in b_{i}}\left|deg\left(b_{i}\right)-deg\left(a\right)\right| & =\sum_{b_{i}\in B}\frac{|b_{i}|-1}{|b_{i}|}min\left(\sum_{a\in b_{i}}deg\left(a\right),\theta \right) \end{array}$$

Combining these three components, the objective function of overall binning scheme cost can be summarized as following, and an optimized binning scheme is found by solving this Minimum Binning Scheme Cost Problem.

(12) $$\begin{array}{cc} \; & \min\;\;\sum_{b_{i}\in B}\left(RC\left(b_{i},u_{d},\theta ,\epsilon_{2},k\right)+AC\left(b_{i},{\bar{v}}_{b},v_{a}\right)+DC\left(b_{i},u_{d},\theta \right)\right)\\ \; & s.t.\;\left\{\begin{array}{c} u_{d},{\bar{v}}_{i},v_{j}\in \left\{0,1\right\}d\in \left[1,\ldots ,\theta \right]\\ i\in [1,\ldots ,r]\\ j\in [1,\ldots ,m]\\ 1\leq k\leq r\leq m\\ \epsilon_{2}=\epsilon /2 \end{array}\right. \end{array}$$

**Algorithm 3** Adaptive Binning Scheme (ABS).**Input:** Local graphs *G*, attribute frequency $\phi^{a}$, attribute-degree distribution $\psi^{d}$, privacy budget ϵ, basic binning $B_{u}$.
**Output:** Optimized binning scheme $B_{o}$ with minimal overall cost.
   1: //compute cost for all possible binning schemes
   2: **for**
B∈B
**do**
   3:     //merge basic bins $a\in B_{u}$ with degree truncation
   4:     **for** $b_{i}\in B$ and $a\in b_{i}$ **do**
   5:         ${\bar{v}}_{i}=1$ if $\exists v_{a}=1$ and $a\in b_{i}$
   6:         $deg\left(b_{i}\right)=min\left(\sum_{a\in b_{i}}deg\left(a\right),\theta \right)$
   7:         $\hat{deg}\left(a\right)=\frac{deg(b_{i})}{|b_{i}|}$
   8:         //compute three cost components for merged bins
   9:         $RC\left(b_{i}\right)=(\frac{1}{1+exp(\epsilon )}(|\theta -max\left(deg\left(b_{i}\right)\right)|-\sum_{a\in b_{i}}|\theta -max\left(deg\left(a\right)\right)\left|\right)+max\left(\sum_{a\in b_{i}}deg\left(a\right)-\theta ,0\right))$
   10:         $AC\left(b_{i}\right)={\bar{v}}_{i}\left(\right|b_{i}\left|-\sum_{a_{j}\in b_{i}}v_{j}\right)$
   11:         $DC\left(b_{i}\right)=\frac{|b_{i}|-1}{|b_{i}|}min\left(\sum_{a\in b_{i}}deg\left(a\right),\theta \right)$
   12:     **end for**
   13: **end for**
   14: //solving the objective function
   15: $B_{o}=argmin_{B\in B}\sum_{b_{i}\in B}\left(RC\left(b_{i}\right)+AC\left(b_{i}\right)+DC\left(b_{i}\right)\right)$
   16: **return**
$B_{o}$


The pseudocode of Adaptive Binning Scheme is presented in Algorithm 3, which mainly computes the overall cost for each possible binning scheme in universal set B and outputs the optimized one. Due to its independence on background knowledge, ABS relies on the estimation of noisy graph data, where each user locally counts the statistics based on uniform binning $B_{u}$ of heterogeneous attributes and randomly perturbs graph data with privacy budget ϵ (Note that uniform binning $B_{u}$ in ABS is alternative and other reasonable binning scheme is applicable). After local private graph being perturbed with binning and truncating processes, data curator correspondingly collects the estimation of binning vectors $\left({\bar{v}}_{1},\ldots ,{\bar{v}}_{i},\ldots ,{\bar{v}}_{r}\right)$ and degrees $deg(b_{i})$ for each bin $b_{i}\in B$, then the overall cost of a binning scheme B∈B is calculated according to Equations (9)–(11). Finally, the optimal binning scheme $B_{o}$ is obtained with dynamic programming by solving Minimum Binning Scheme Cost Problem in Equation (12). Because ABS is executed on the server side, it brings no computational overhead to local devices.

### 5.2. Randomization Optimization

On the basis of aforementioned optimal binning scheme $B_{o}$ generated by ABS, minimal overall binning cost is achieved when aggregating heterogeneous graph data. One direct benefit is that ABS generally scales the domain size of graph attributes from |A|=m down to $|B_{o}|=r$ and reduces the storage and communication burden reduction on local devices. Furthermore, this subsection continues to provide optimizing strategies for user-side local randomization of the PrivHG mechanism, which mainly contains two parts sampling subset size and fake degree generation.

Sampling subset size. During the data randomization component of PrivHG, *k*-sized subset of attribute and degree data are sampled, randomized and contributed, with privacy budget ϵ split among these *k* pairs of data. Therefore, altering *k* strictly affects estimation accuracy, budget usage and communication overhead. Under the circumstance that privacy budget and communication resources are sufficient, theoretically optimal parameter $k_{o}$ can be selected by taking estimation accuracy into account and minimizing the variance.

$$k_{o}=argmin\left(\sum_{j\in [1,r]}Var\left[\phi^{j}\right]+\sum_{j\in [1,r],t\in [1,\theta ]}Var\left[\psi_{j}^{t}\right]\right)$$

Although the derivation of a closed-form optimal $k_{o}$ is almost impossible, due to the complexity of computing variances $Var[\phi^{j}]$ and $Var[\psi_{j}^{t}]$, $k_{o}$ can still be selected from thorough computation based on public parameters. Before distributing parameters for PrivHG, data curator first computes all $Var\left[PrivHG\right]=\sum_{j\in [1,r]}Var\left[\phi^{j}\right]+\sum_{j\in [1,r],t\in [1,\theta ]}Var\left[\psi_{j}^{t}\right]$ of every possible k∈[1,m], and select one with minimal variance as the $k_{o}$. However, under circumstances where privacy budget or communication resources are limited, a large *k* will bring about much difficulties in practical execution of PrivHG. Therefore, a feasible approach is to sacrifice a minor proportion of estimation accuracy in exchange for a communication overhead reduction and overall privacy budget utilization by fixing k=1, which is denoted as $k_{e}$-PrivHG.

Deployment of PrivHG on heterogeneous graph with $k_{o}$ or $k_{e}$ is pretty empirical. Hardware resource constraint is a viable standard as stated above. Another feasible standard is based on the sparsity of heterogeneous graph data, because the performance of randomization relies on data sparsity and practical heterogeneous graphs may have pretty different data distributions. When perturbing sparse graph data, the aggregation and estimation are usually inaccurate, so the optimal $k_{o}$-PrivHG is picked to improve the estimation accuracy. When perturbing dense graph data, diminution of sampling size with $k_{e}$-PrivHG is reasonable, which reduces communication overhead and utilizes privacy budget as a whole. The general principle is that deploying $k_{o}$-PrivHG on small and sparse graph data and picking $k_{e}$-PrivHG otherwise. In the experiment section, We reasonably pick these two mechanisms for different datasets, and leave the fine-grained contextual-dependent selection of *k*-PrivHG for heterogeneous graph as future work.

Fake degree generation. Due to the intrinsic correlation within heterogeneous graph data, the perturbation of attributes and degrees should remain correlated, otherwise the information loss results in inaccurate estimates. PrivHG ensures that degree randomization follows the result of attribute randomization. Specifically, there are four possible cases for randomizing indicating bit of merged bins ${\bar{v}}_{b}\to {\tilde{v}}_{b}$: 1→0,1→1,0→0,0→1. When an indicating bit of merged bin is perturbed to ${\tilde{v}}_{b}=0$ (${\bar{v}}_{b}=1$ or ${\bar{v}}_{b}=0$), the corresponding aggregated binning degree deg(b) should be set as 0 (equivalent to set degree vector ${\tilde{u}}^{b}=\left[0,\ldots ,0\right]$) regardless of perturbed degree value, otherwise the correlation between them will be violated. When ${\bar{v}}_{b}=1$ is randomized as ${\tilde{v}}_{b}=1$, degree bit ${\tilde{u}}_{deg(b)}$ is normally randomized and retained ( The corresponding randomization in Algorithm 4 is achieved by multiplying two randomized vectors ${\tilde{u}}^{b}={\tilde{u}}^{b}\cdot {\tilde{v}}_{b}$). In the case of ${\bar{v}}_{b}=0$ and ${\tilde{v}}_{b}=1$, the corresponding aggregated binning degree needs to satisfy deg(b)≠0, but local user has no related degree data to be randomized, therefore PrivAG randomly generates a fake degree from [1,θ] as deg(b), which skews the estimation of attributed degree distribution. With the help of Algorithm 3, the fake degree generation is further refined in PrivHG, therefore neutralizing the skewing effect on the estimation. For ${\bar{v}}_{b}=0$ being randomized as ${\tilde{v}}_{b}=1$, the generation range of fake degree is scaled to [min(deg(b)),max(deg(b))] instead of [1,θ] to prevent outliers being generated, and the generation probability is set to the estimated frequency of degrees $\psi_{j}^{t}$ instead of equal probabilities $\frac{1}{\theta }$ to reduce the skewness. deg(b) and $\psi_{j}^{t}$ can be inferred from the postprocessed statistics of ABS without violating ϵ-ALDP.

### 5.3. Estimation Optimization

This subsection corresponds to the last step in Figure 2 and provides optimization techniques for server-side aggregation and estimation. Similar to Algorithm 1, PrivHG aggregates bit 1s in perturbed attribute and degree vectors, and makes an unbiased estimation based on the aggregation. Since the estimated statistics should follow the characteristic of heterogeneous graph data, two postprocessing approaches are further proposed in PrivHG to filter out the aggregated outliers and correct the final statistical estimation.

Attribute Bin Frequency Estimation. The probabilities of Equations (3) and (4) are critical to make an unbiased estimation of attribute distribution $\phi^{a}$. Since ABS resizes the domain size through aggregating attributes into bins, then the two binning randomization probabilities of ${\hat{p}}_{b}=Pr\left[{\hat{v}}_{b}=1|{\bar{v}}_{b}=1\right]$, and ${\hat{q}}_{b}=Pr\left[{\hat{v}}_{b}=1|{\bar{v}}_{b}=0\right]$ are derived as follows.

p^b=exp(ϵ1)r+ℓ′−1k−1rk+exp(ϵ1)(r+ℓ′k−rk)q^b=r−1k−1+exp(ϵ1)(r+ℓ′−1k−1−r−1k−1)rk+exp(ϵ1)(r+ℓ′k−rk)

Based on the above probabilities, the expected counts of aggregated bins ${\hat{C}}_{b}$ is denoted as:

(13) $$E\left[{\hat{c}}_{b}\right]=E\left[\#\left\{i|{\hat{v}}_{b}^{i}=1,i\in \left[1,n\right],b\in \left[1,r\right]\right\}\right]=\phi^{b}n{\hat{p}}_{b}+\left(1-\phi^{b}\right)n{\hat{q}}_{b}$$

Then unbiased estimation of attribute bin frequency is:

(14) $$\phi^{b}=\frac{{\hat{c}}_{b}-n{\hat{q}}_{b}}{n({\hat{p}}_{b}-{\hat{q}}_{b})}$$

The common way to optimize frequency estimation like $\phi^{b}$ is to clip it with range [0,1]. In PrivHG, a better lower bound is given based on the characteristic of heterogeneous graph. Assume an extreme case, where there is only one edge $e_{xy}$ corresponding to a merged bin *b* in the whole heterogeneous graph, then at least two nodes *x* and *y* report attribute data ${\hat{v}}_{b}^{x}=1$ and ${\hat{v}}_{b}^{y}=1$, and the least aggregated bits count ${\hat{c}}_{b}$ for each merged bin *b* is 2, therefore the lower bound of $\phi^{b}$ should be $\frac{2}{n}$. The estimation of attribute distribution ${\hat{\phi }}^{b}$ is derived by clipping $\phi^{b}$ with range $\left[\frac{2}{n},1\right]$.

Binned Degree Frequency Estimation. Similar to the estimation in Section 4, binned degrees are estimated based on the aggregated bins in $B_{o}$. The expected counts of binned degree ${\hat{d}}_{b}^{t}$ is derived as follows, where ${\hat{p}}_{d}=\frac{1}{2}$ and ${\hat{q}}_{d}=\frac{1}{exp(\epsilon_{2}/k)+1}$

(15) $$E\left[{\hat{d}}_{b}^{t}\right]=E\left[\#\left\{i|{\hat{u}}_{b}^{t}\left(i\right)=1\right\}\right]=\psi_{b}^{t}n\phi^{b}{\hat{p}}_{d}+\left(1-\psi_{b}^{t}\right)n\phi^{b}{\hat{q}}_{d}$$

Then unbiased estimation of binned degree frequency is:

(16) $$\psi_{b}^{t}=\frac{{\hat{d}}_{b}^{t}-n\phi^{b}{\hat{q}}_{d}}{n\phi^{b}\left({\hat{p}}_{d}-{\hat{q}}_{d}\right)}$$

Since the attributed degree distribution cannot be negative, the ${\hat{\psi }}_{b}^{t}$ is first clipped with [0,1] for each bin in $B=[b_{1},\ldots ,b_{r}]$ to eliminate negative influences of outliers. Then the estimations are further corrected based on the nature of graph data. Considering the one characteristic of graph edges that the total number of edges have an upper bound $\frac{n(n-1)}{2}$, which is the edge number of complete graph with n nodes. Similarly in the context of PrivHG, the maximum degree is truncated as θ for each bin in *B*, therefore the total number of edges cannot exceed that of θ-complete graph with $n_{j}$ nodes, where $n_{j}$ is the number of binned nodes with $b_{j}\in B$ and can be derived by corresponding estimated attribute bin frequency as $n\phi^{b}$, then the upper bound of total edges is $\frac{n\phi^{b}\theta }{2}$. Since the lower bound of total edges is 1, the total edges $\sum_{t\in [1,\theta ]}tn_{bt}\psi_{b}^{t}$ for each bin b∈B is bounded as:

(17) $$1\leq \frac{\sum_{t\in [1,\theta ]}tn_{bt}\psi_{b}^{t}}{2}\leq \frac{n\phi^{b}\theta }{2}$$

Given that 2<θ, the refined estimation of binned degree frequency is derived by substituting Equation (17) into (16):

(18) $${\hat{\psi }}_{b}^{t}=\frac{{\hat{d}}_{b}^{t}-n\phi^{b}{\hat{q}}_{d}}{tn\phi^{b}\left({\hat{p}}_{d}-{\hat{q}}_{d}\right)}\cdot max\left(\frac{2}{\sum_{t\in [1,\theta ]}\psi_{b}^{t}t},1\right)\cdot min\left(\frac{\theta }{\sum_{t\in [1,\theta ]}\psi_{b}^{t}t},1\right)$$

### 5.4. PrivHG Mechanism

In this subsection, we present the overall PrivHG mechanism based on aforementioned building blocks. The detailed pseudocode of PrivHG is listed in Algorithm 4.
**Algorithm 4** PrivHG.**Input:** local heterogeneous graphs *G*, privacy budget ϵ.
**Output:** attribute frequency ${\hat{\phi }}^{a}$, attribute-degree distribution ${\hat{\psi }}^{d}$.
   1: //user-side randomization with basic binning
   2: $B_{u}=\left\{b_{i}=\left(1+\left(i-1\right)\delta ,1+i\delta \right)|i\in \left(1,r\right),\delta =\frac{w-1}{r}\right\}$
   3: $\bar{G^{\prime }}\leftarrow pre-process\left(G^{\prime },B_{u}\right)$
   4: $\phi^{a},\psi^{d}\leftarrow PrivAG\left(\bar{G^{\prime }}\right)$
   5: //server-side optimal binning scheme selection
   6: $B_{u}^{\prime }\leftarrow B_{u}\cup A_{c}$
   7: $B_{o}\leftarrow ABS\left(\bar{G^{\prime }},\phi^{a},\psi^{d},\epsilon_{2}/k,B_{u}^{\prime }\right)$
   8: redistribute parameters $B_{o}$, $k_{e}$ or $k_{o}$
   9: //user-side randomization with optimal binning
   10: ${\bar{G}}^{\prime \prime }\leftarrow pre-process\left(G^{\prime \prime },B_{o}\right)$
   11: ${\hat{v}}_{b},{\hat{u}}_{b}^{t}\leftarrow DRC\left({\bar{G}}^{\prime \prime }\right)$
   12: //server-side estimation with correction
   13: $c_{j}\leftarrow count\left({\hat{v}}_{b}\right)$
   14: $d_{b}^{t}\leftarrow count\left({\hat{u}}_{b}^{t}\right)$
   15: **for**
b∈[1,r]
**do**
   16:     estimate attribute bin frequency: $\phi^{b}=\frac{{\hat{c}}_{b}-n{\hat{q}}_{b}}{n({\hat{p}}_{b}-{\hat{q}}_{b})}$
   17:     clip $\phi^{b}$ with $\left[\frac{2}{n},1\right]$
   18: **end for**
   19: **for**
b∈[1,r]andt∈[1,θ]
**do**
   20:     estimate binned degree frequency with refinement as:                ${\hat{\psi }}_{b}^{t}=\frac{{\hat{d}}_{b}^{t}-n{\hat{\phi }}^{b}{\hat{q}}_{d}}{tn{\hat{\phi }}^{b}\left({\hat{p}}_{d}-{\hat{q}}_{d}\right)}\cdot min\left(\frac{\theta }{\sum_{t\in [1,\theta ]}\psi_{b}^{t}t},1\right)$
   21:     clip ${\hat{\psi }}_{b}^{t}$ with $\left[\frac{1}{n{\hat{\phi }}^{b}},1\right]$
   22: **end for**
   23: **return**
${\hat{\phi }}^{a}$ and ${\hat{\psi }}^{d}$


To elaborate, PrivHG mechanism first generalizes Algorithm 3 as a fundamental subtask to deal with mixed-attributed data in local graph. There are two conventional approaches regarding the execution of subtasks, one is to divide the privacy budget ϵ as several parts for each subtask to execute on the complete data set, and the other is to divide the user data while each subtask consuming the complete privacy budget ϵ to execute on a portion of data set. The former approach of dividing privacy budget ϵ leads to inaccurate estimations especially for heterogeneous graph. In contrast, executing subtasks separately on divided data sets utilizes the full privacy budget and comparatively reduces the overall error, which has been adopted by several recent studies and is also applied in our PrivHG mechanism. The heterogeneous graph *G* is divided as two groups $G^{\prime }$ and $G^{\prime \prime }$, where ABS subtask is executed on $G^{\prime }$ to derive the optimized binning $B_{o}$ and $B_{o}$ is employed on $G^{\prime \prime }$ to make an unbiased estimation.

During the initialization phase of PrivHG, numerical-attributed data of $G^{\prime }$ is divided by a basic uniform binning $B_{u}$ (other schemes like geometric binning is alternative), and each interval is treated equally as the categorical attributes. Based on $B_{u}$, Algorithm 2 aggregates statistics of preprocessed $G^{\prime }$. Then on the server side, ABS outputs generalized optimal binning scheme $B_{o}$ for mixed attributes by enlarging the input domain as $B_{u}\cup A_{c}$, which is the union set of numerical-attributed and categorical-attributed data. Generalized ABS makes no assumption about the attribute type, and solely optimizes Equation (12) based on the degrees of each merged bin $b_{i}\in B_{u}^{\prime }$. In order to further mitigate the influences brought by noisy degree outliers, we choose to remove 5% marginal data when practically executing ABS in this paper. On the one hand, these marginal values may be biased outliers that are randomly generated from vacant vector bits, and the estimation error will be reduced if removing these outliers. On the other hand, even actual marginal data may account for a relatively small proportion of whole data due to the data distribution of graph, which have a minor impact on the results. In the following phase, the optimal binning scheme $B_{o}$ and necessary parameters are informed to the other subset of heterogeneous graph $G^{\prime \prime }$, and each user preprocesses and randomizes local data as Algorithm 1, in which fake degree generation is calibrated as stated in Section 5.2 instead of randomly selected. Finally, these randomized vectors are aggregated by the server, then unbiased estimations with correction and refinement are made according to Section 5.3.

According to composition and postprocessing theorems, aggregating the two statistics of heterogeneous graph under PrivHG mechanism satisfies ϵ-attributewise local differential privacy, and proof of which is omitted due to the triviality.

## 6. Experimental Evaluation

In this section, we evaluate the estimation performance of proposed PrivHG and comparison mechanisms on extensive scenarios.

Evaluated Mechanisms. For attribute and degree distribution estimation on categoricalattributed graph, we utilize the generalized randomized response (GRR) mechanism to perturb local data as in [36,37], which is compared with PrivAG and PrivHG (PrivHG executes ABS solely on categorical-attributed data). For estimation on heterogeneous graph with mixed-attributed data, we combine GRR and basic binning scheme (both uniform binning $B_{u}$ and geometric binning $B_{g}$ schemes) to uniformly perturb heterogeneous data, which is denoted as BGRR, and PrivAG is also tentatively extended to heterogeneous graph with basic binning scheme. These two mechanisms are compared with PrivHG.

General Setting. The experiments are implemented on various synthetic Erdos–Renyi random graphs [38], which gives a general simulation about the real-world datasets. To be specific, we separately generate graphs with different attributes based on parameter *m*, *w* and *n*, and merge them together as a heterogeneous graph in each experiment epoch, the number of users/nods *n* is set to 5000, the categorical attribute domain size *m* ranges from 8 to 32, the numerical attribute range bound *w* varies from 10 to 20. To simulate different attributed data sparsity of heterogeneous graphs, the maximum number of synthetic edges for each attribute follows the Uniform/Gaussian distribution (μ=0 and σ=10). During the data preprocess part, truncation parameter θ range from 10 to 50, and the privacy budget ϵ ranges from 0.005 to 5.0, with $\epsilon_{1}=\epsilon_{2}=\epsilon /2$. Each setting of the experiments runs 100 times, and the result are average of these experiments.

Performance Metrics. The performance of attribute and degree distribution estimation is evaluated by MSE ($\ell_{2}$-norm error):

(19) $$∥{\hat{\phi }}^{a}-\phi^{a}{∥}_{2}=E\left[\sqrt{{∥{\hat{\phi }}^{a}-\phi^{a}∥}^{2}}\right],\;{∥{\hat{\psi }}^{d}-\psi^{d}∥}_{2}=E\left[\sqrt{{∥{\hat{\psi }}^{d}-\psi^{d}∥}^{2}}\right]$$

where $\phi^{a}$ and $\psi^{d}$ (resp. ${\hat{\phi }}^{a}$ and ${\hat{\psi }}^{d}$) are the true distribution of attributes and attributedegrees (resp. estimated).

**Figure 3:**
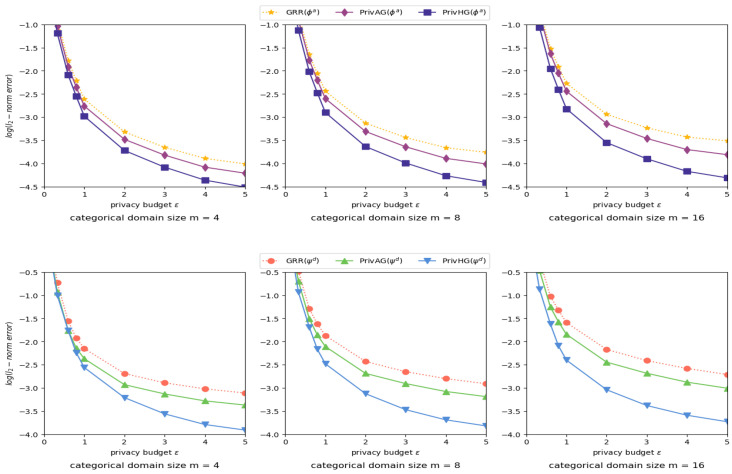
Categorical-attributed graph aggregation with different attribute domain size.

Influence of categorical attribute domain size. Figure 3 shows the estimation error of categorical-attributed graph aggregation, with different categorical attribute domain size *m* and privacy budget ϵ settings. It can be observed from the figures that the estimation error reduction grows larger as the domain size *m* increases, and PrivHG is less affected by domain size than other two mechanisms. In most settings, PrivHG outperforms GRR and PrivAG on both attribute frequency and attribute-degree distribution estimation.

**Figure 4:**
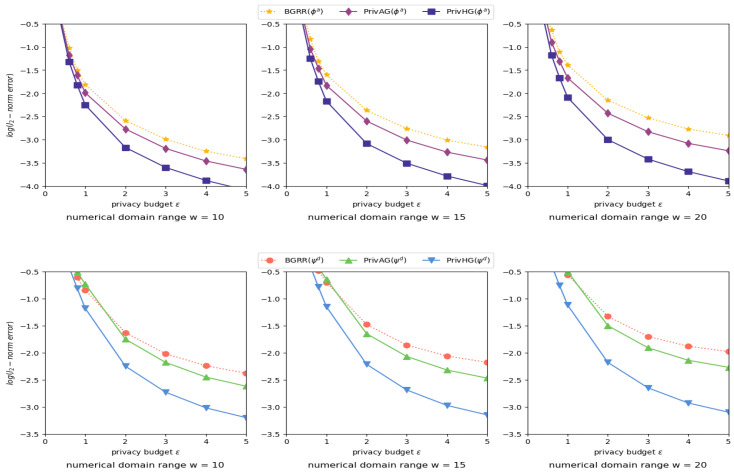
Heterogeneous graph aggregation with different numerical attribute range.

Influence of numerical attribute domain range. Figure 4 shows the results of heterogeneous graph aggregation with varied numerical attribute domain *w* and fixed categorical attribute domain size m=32, where BGRR and PrivAG apply uniform binning scheme to deal with numerical-attributed data. PrivHG outperforms BGRR and PrivAG in most settings. As *w* increases, the reduction of attribute frequency estimation error among three mechanisms is minor, while the degree distribution estimation error of PrivHG decreases faster than other two mechanisms.

**Figure 5:**
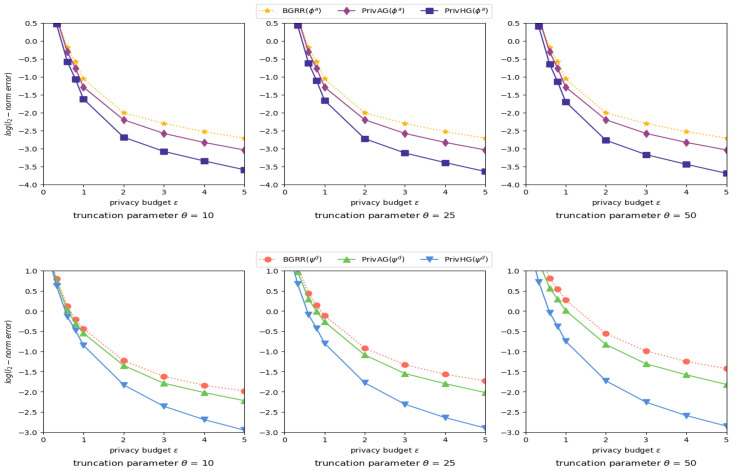
Heterogeneous graph aggregation with different truncation parameter.

Influence of truncation parameter. Figure 5 shows the results of heterogeneous graph aggregation with different truncation parameter θ and privacy budget ϵ settings. When θ grows larger, the degree estimation accuracy of BGRR and PrivAG degrades a lot due to excessive vacant bits being randomized as noises, but results of PrivHG have a relatively significant improvement. The error reduction of attribute estimation is slightly affected by truncation parameter θ between PrivHG and other mechanisms. In most cases, PrivHG outperforms BGRR and PrivAG on distribution estimation.

**Figure 6:**
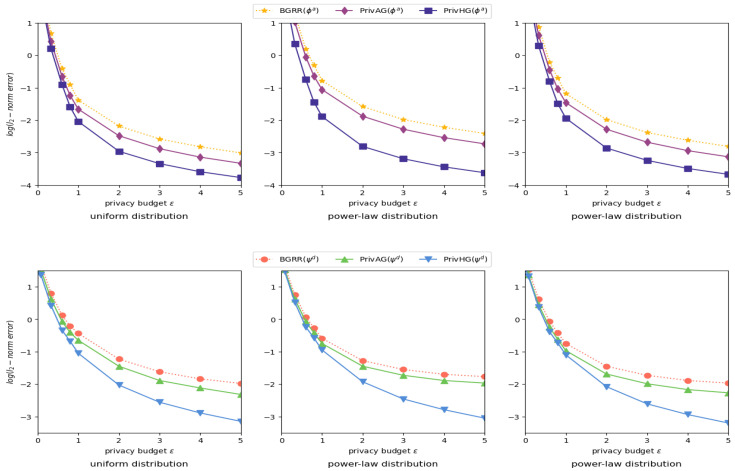
Heterogeneous graph aggregation with uniform binning and Uniform distribution (**left**), uniform binning and Gaussian distribution (**middle**), geometric binning and Gaussian distribution (**right**).

Influence of data distribution and binning scheme. Figure 6 shows the results of heterogeneous graph aggregation with different data distribution/sparsity and various binning schemes. As can be summarized from these figures, the estimation error reduction between PrivHG and other two mechanisms is rather noticeable when the data distribution and binning scheme are dissimilar, which could be due to the reliance of BGRR and PrivAG on the consistency of intrinsic graph data distribution and binning scheme. In most settings, PrivHG is stable and outperforms BGRR and PrivAG on both attribute frequency and attribute-degree distribution estimation.

In summary, above experiments show that it is feasible to preserve privacy for heterogeneous graph data under ϵ-ALDP with high fidelity, and PrivHG mechanism significantly outperforms baseline mechanisms on statistical results by reducing 43% estimation error in average. Furthermore, the proposed PrivHG mechanism is well suited to deal with various heterogeneous graphs and does not rely on specific data sparsity or attribute binning scheme.

## 7. Related Work

The de facto Differential Privacy (DP) notion have form the theoretical basis of a considerable amount of research literature for past decade. By assuming a centralized and trustworthy data curator [15,39], several fundamental mechanisms achieving differential privacy constraint have been proposed to deal with numerical and categorical data, including Laplace mechanism in [6] and Exponential mechanism in [7]. However, under the gradually increasing risk of adversaries prying into personal privacy and the growing expectation to keep private data on personal devices, the emphasis of privacy-preservation studies has shifted from centralized settings to local settings.

Local Differential Privacy [40] ensures that private data are perturbed locally on each user’s devices, thus avoiding the reliance on trustworthiness of data curator and broadening the applicable scenario of DP. A variety of studies protecting local differential privacy have been constantly emerging. The pioneer study of Randomized Response, which was proposed by [33], satisfies local differential privacy guarantee well, and many following studies are built on it. Its variants play an important role in the categorical data domain [8,11,14,41,42,43]. Later on, the study of LDP is expanded to more promising fields. Ref. [34] summarizes the characteristics of existing mechanisms and proposes OUE and OLH to better adapt to various novel scenarios. Ref. [20] presents a twophase framework for aggregating set-valued data under local differential privacy, and [19] proposes a generalized mechanism PrivSet to perturb a sampled subset of set-valued data domain and provides optimized estimation guarantee. As for numerical data, Ref. [44] utilizes square wave and smoothing mechanism to maximum the estimation expectation of numerical data distribution, and [45] proposes an adaptive hierarchy-based mechanism to privately answer range query. Beyond the single datatype, Ref. [24] designs an iterative mechanism PrivKVM to locally privately collect key-valued data, and retain the correlation between key-value pairs. Ref. [25] optimizes the estimation accuracy and communication cost of PrivKVM mechanism. These studies offer powerful tools for tackling our problem.

Due to its intrinsic complexity, preserving private graph requires additional concerns. According to the variations of privacy granularity, differential privacy for graph data can be generally divided into two groups [46]: node-based and edge-based, which provide protection either on edge-level privacy or on node-level privacy. Based on different privacy granularity, various problems are studied, such as publishing private degree frequency [47,48], aggregating graphic statistics [49,50] and synthetic graph generation [51,52]. Recently, graph data aggregation mechanisms under LDP constraint have been studied. Ref. [53] manages to aggregate node degrees and weighted edges based on 1-neighborhood graph in the local setting. By defining neighboring clusters, collecting neighboring degrees and refining the clusters, Ref. [30] proposes an iterative graph generation framework LDPGen to generate synthetic graphs. Ref. [31] introduces a novel privacy notion DDP for social networks, and provides a multiphased framework to aggregate subgraph statistics. Ref. [32] presents a graph generative framework AsgLDP, capturing node features and generating node-attributed graph. Ref. [37] extends the research fields to multiplex graphs and proposes to locally privately estimate clustering coefficients on them. However, these research studies of preserving local private graph data mainly focus on edge-based LDP for graph and neither of them provides stronger privacy guarantee while aggregating heterogeneous graph data.

## 8. Conclusions

In this paper, we study the heterogeneous graph aggregation with a unified, efficient and effective PrivHG mechanism under local differential privacy. We combine characteristics of two conventional LDP variants and propose a fine-grained privacy definition for locally private heterogeneous graph, which generally provides stronger privacy guarantee than edge-based LDP and higher estimation accuracy than node-based LDP. We design a unified mechanism PrivHG to aggregate two statistics of heterogeneous graph while protecting the fine-grained attributewise local differential privacy. Furthermore, we propose several optimization techniques for reducing the computation costs and estimation errors of PrivHG mechanism in practical application. The effectiveness and efficiency of the PrivHG mechanism are validated through extensive experiments.

We will investigate the application of PrivHG with other graph analysis tasks and extend the perturbation mechanisms for other correlated and heterogeneous data types for future work.

## Figures and Tables

**Table 1:** Notations.

Symbol	Meaning
G(V,E,A)	attributed graph
$G^{i}$	local graph of *i*-th user
$A^{i}$	possessed attribute set of *i*-th user
*m*	categorical attribute domain size $|A_{c}|=m$
*w*	numerical attribute domain size $|A_{n}|=w$
*ℓ*	maximum attributes each user have $|A^{i}|\leq \ell$
$a_{j}$	the *j*th attribute from A
$deg^{i}\left(a_{j}\right)$	number of edges in $G^{i}$ have attribute $a_{j}\in A$
θ	maximum degree bound
$v_{a}$	attribute vector
$u_{d}$	degree vectors
$\phi^{a}$	frequency of attribute *a*
$\psi^{d}$	degree distribution of *d*

## Data Availability

Not applicable.
